# A human-like glutaminase-free asparaginase is highly efficacious in ASNS^low^ leukemia and solid cancer mouse xenograft models

**DOI:** 10.1016/j.canlet.2024.217404

**Published:** 2024-12-19

**Authors:** Maaike Van Trimpont, Amanda M. Schalk, Kenneth Hofkens, Evelien Peeters, Sara T’Sas, Katrien Vandemeulebroecke, Ying Su, Ashley De Loera, Alyssa Garcia, Hui Chen, Tim Lammens, Pieter Van Vlierberghe, Steven Goossens, Arnon Lavie

**Affiliations:** a Cancer Research Institute Ghent (CRIG), Ghent, Belgium; b Department of Diagnostic Sciences, Ghent University, Ghent, Belgium; c Department of Biochemistry and Molecular Genetics, University of Illinois at Chicago, Chicago, USA; d Enzyme By Design Inc., Chicago, USA; e Department of Pediatric Hematology-Oncology and Stem Cell Transplantation, Ghent University Hospital, Ghent, Belgium; f Department of Internal Medicine and Pediatrics, Ghent University, Ghent, Belgium; g Director, Mass Spectrometry Core, Research Resources Center, University of Illinois at Chicago, Chicago, USA; h Department of Biomolecular Medicine, Ghent University, Ghent, Belgium; i Research Biologist, Biological Science Research and Development, Department of Veterans Affairs Medical Center, Chicago, IL, USA

**Keywords:** (Max 7), Asparagine synthetase, Acute lymphoblastic leukemia, ASNS^low^ solid tumors, Liver cancer, Melanoma

## Abstract

L-asparaginase (L-ASNase) is crucial in treating pediatric acute lymphoblastic leukemia (ALL), but its use is hampered by side effects from the immunogenicity and L-glutaminase (L-GLNase) co-activity of FDA-approved bacterial L-ASNases, often leading to treatment discontinuation and poor outcomes. The toxicity of these L-ASNases makes them especially challenging to use in adult cancer patients. To overcome these issues, we developed EBD-200, a humanized guinea pig L-ASNase with low Km and no L-GLNase activity, eliminating glutamine-related toxicity. EBD-200 showed comparable anti-cancer effects to PEGylated L-ASNase in ASNS^low^ ALL, melanoma and liver cancer models, with improved tolerability. Its potent anti-cancer efficacy and enhanced safety profile suggest that EBD-200 could benefit ALL patients and broaden treatment options for ASNS^low^ solid cancers.

## 1. Introduction

L-asparaginase (L-ASNase) stands as a pivotal enzyme drug in the treatment of acute lymphoblastic leukemia (ALL), exhibiting remarkable efficacy [1,2]. Its mechanism of action involves the depletion of extracellular asparagine, by hydrolyzing it into aspartate and ammonia [3]. Asparagine, although non-essential for most cells due to endogenous synthesis facilitated by asparagine synthetase (ASNS), becomes indispensable for cells lacking ASNS expression, a characteristic often observed in ALL cells [4–6]. Historically, clinically approved L-ASNases have been exclusively of bacterial origin, primarily derived from *Escherichia coli* (naked version previously marketed in the US as Elspar; PEGylated versions marketed as Oncaspar and Asparlas) [7] or *Erwinia chrysanthemi* (originally marketed as Erwinaze; now as Rylaze in the US, Enrylaze in the EU) [2,8–10]. The selection of these bacterial L-ASNases stemmed from their superior kinetic properties. Specifically, their low Km for asparagine is a requisite for efficient blood asparagine depletion [11]. In the last five decades, the significance of L-ASNases in ALL treatment has been consistently observed. Whether through direct comparisons between patients who received L-ASNase versus those who did not, or evaluations of the efficacy of intensified asparaginase regimens, superior therapeutic outcomes consistently emerge in patients receiving maximal asparaginase therapy [12–14].

However, the bacterial origin of these therapeutic enzymes renders them prone to immunogenic responses [15–17], necessitating strategies such as PEGylation to mitigate hypersensitivity reactions [18,19]. Originally, PEGylation reduced the occurrence of hypersensitivity reactions [20,21]. However, likely due to the widespread use of PEG in consumer goods, in over 25 % of the healthy population pre-existing anti-PEG antibodies can be detected [22,23]. As a result, the PEG moiety itself may induce immunogenic responses or silent inactivation against PEG-ASNase [24,25] and 10–20 % of ALL patients require a shift to Erwinaze/Rylaze/Enrylaze [17,26–28]. An additional advantage of PEGylation is prolonged drug persistence *in vivo*, which enables less frequent dosing regimens [19,29,30]. This allowed treatment to shift from 3 times per week for the naked bacterial enzymes to once every 2–3 weeks with the PEGylated versions, Oncaspar and Asparlas, respectively. Despite these advancements, a subset of ALL patients still fails to complete their L-ASNase treatment course due to immunogenicity [2]. Moreover, bacterial L-ASNases exhibit glutaminase (L-GLNase) co-activity, whose role in clinical efficacy and toxicity remains debated, but has been postulated to drive adverse effects, including pancreatitis and coagulation abnormalities [2,31–33].

Accumulating evidence suggests that L-ASNases could also have clinical potential for the treatment of other aggressive subtypes of cancer, including acute myeloid leukemia (AML) [34–36], ovarian cancer [37–39], pancreatic cancer [40–42], hepatocellular carcinoma (HCC) [43–45], colorectal (CRC) [46–48] and metastatic breast cancer [2,49]. However, because of the tolerability and toxicity issues associated with extended/long term use of these enzymes observed in different clinical studies [39,42], the clinical potential of L-ASNase for the treatment of these solid tumors has not been fully pursued. Therefore, the availability of a L-ASNase variant with less side-effects would not only be beneficial for ALL patients but could also provide extra therapeutic opportunities for solid cancer patients.

Notably, the guinea pig L-ASNase offers a potential avenue for reduced immunogenicity due to its increased sequence identity with the human homolog as well as fewer side effects, owing to its inherent glutaminase-free nature. Building upon our previous findings that demonstrated equivalent *in vivo* efficacy and better tolerability of a L-GLNase-low Erwinaze variant [50,51], we developed a humanized L-ASNase variant, termed EBD-200, derived from the guinea pig L-ASNase [52]. Through a process of humanization and identification of potential T cell epitopes, we aimed to enhance immune tolerance while preserving efficacy. In this study, we show that our L-GLNase-free humanized EBD-200 can match the efficacy of its bacterial counterpart PEG-ASNase in ASNS-deficient leukemia, melanoma and liver cancer mice xenograft models. Notably, EBD-200 shows a more favorable toxicity profile, allowing long term use of our novel L-ASNase which makes it highly clinically relevant.

In summary, our findings demonstrate the potential of EBD-200 as a highly effective and tolerable alternative to bacterial L-ASNases, paving the way for broader utilization in both pediatric and adult cancer patients, including those with ASNS-deficient solid tumors.

## 2. Methods

### 2.1. Engineering of EBD-200

EBD-200 is based on a two-step humanization process of the N-terminal asparaginase domain of *Cavia porcellus* (guinea pig) asparaginase UniProt entry H0W0T5. In the first step, the crystal structure of the full-length guinea pig asparaginase [52] revealed truncation at around reside ~359 (PDB codes 4R8K and 4R8L). These and structures of catalytic mutations (PDB codes 5DNC, 5DND, 5DNE) were used for a rational structure-guided design whereby humanization of surface residues via PCR mutagenesis was conducted on the guinea pig asparaginase construct truncated at residue 359 and comprising an additional 10 amino acids of human asparaginase sequence (UniProt entry Q86U10) resulting in a construct called EBD-100. In the next step, fifty EBD-100 peptides of overlapping sequence that contained non-human residues were generated with >95 % purity and tested for immunogenicity potential in ProImmune’s ProMap Immunogenicity System T cell proliferation assay to generate data to enable the *in vitro* assessment of potential antigenicity of these peptides. The assay was performed by testing the peptides against a panel of peripheral blood mononuclear cell (PBMC) samples from 40 healthy donors, where isolation of the PBMC from donor blood samples includes a CD8^+^ T cell depletion step to eliminate CD8^+^ responses from the analysis. The PBMC panel was selected so that HLA class II alleles known to be highly expressed in the global population were well represented. PBMC were used to provide both T cells and antigen presenting cells (APC) in the assay. Cultures were set up in multi-well plates. Each peptide was tested at a final assay concentration of 5 μM per well. Cells were labeled with the cell dye carboxyfluorescein succinimidyl ester (CFSE) prior to incubation with test peptides. Each test peptide was cultured with each of the donor PBMC samples in six replicate wells. Each plate included six unstimulated control wells. Reference antigens comprising known MHC class II epitopes were used in this study. After 7 days incubation with peptides or control proteins, cells were stained with anti-CD4 antibody, then washed and fixed for flow cytometric analysis. Proliferation was determined by measuring a decrease in CFSE intensity. Detection of proliferation of CD4^+^ T cells was performed by labeling cells with CFSE and co-staining with anti-human CD4 antibody. Data obtained by flow cytometric evaluation of cell samples was analyzed using FlowJo Software (Tree Star, Inc.). The number of CFSE-dim (i.e. proliferating) cells was determined for each sample in six replicate wells. Counts for the CD4^+^ CFSE dim population in each sample were expressed as a proportion of the total CD4^+^ population. The six replicate values were used to calculate Percentage Stimulation above Background (proportion of CD4^+^ CFSE dim cells with antigen stimulation minus proportion of CD4^+^ CFSE dim cells without antigen stimulation). A mean and standard error of the mean (SEM) of the six values was calculated for each sample. A one-way analysis of variance (ANOVA) was also performed on the data to determine (to a significance level of p ≤ 0.05) whether the CFSE dim population size obtained from each peptide differs significantly from the unstimulated control. A response that has Percentage Stimulation above Background ≥0.5 % and is also two standard errors greater than background (SEM = 2), is considered positive. Background threshold values are based on data from unstimulated cells for which a maximum acceptable resting proliferation threshold is established. EBD-200 was generated through PCR mutagenesis of the non-human residues in peptides that fit these criteria for being a potential T cell epitope to the corresponding residues in the human homolog.

### 2.2. Expression and purification of EBD-200

The synthetic gene (Genscript) for guinea pig asparaginase codon-optimized for expression in *E. coli* was subcloned into a His6-SUMO-pET14b (original pET14b from EMD Millipore) vector (where the His6 tag is followed by the yeast protein SUMO (small ubiquitin modifier, Smt3p) expression vector using NdeI and BamHI restriction sites at the 5′ and 3′ ends, respectively. The His6-SUMO-EBD-200-pET14b plasmid was transformed into *E. coli* C41(DE3) cells (a derivative of BL21(DE3)) for expression. Starter cultures of the plasmids were grown overnight and inoculated into 2 YT media at a ratio of 1:100. The cells were grown at 37 °C to an optical density of 0.6–0.8, and overexpression was induced with isopropyl β-d-1-thiogalactopyranoside (IPTG) at a final concentration of 0.3 mM. Growth continued at 18 °C overnight, at which point the cells were harvested by centrifugation, and the pellets were frozen at −20 °C. For purification, the cell pellets were thawed, resuspended in lysis buffer (25 mm Tris-HCl, pH 7.9, 500 mm NaCl, 10 mM MgCl_2_, 20 mm imidazole, 10 % glycerol, 1 % Triton X-100, 1 mm PMSF), and disrupted by sonication. The lysate was cleared by ultracentrifugation, and the supernatant was loaded onto an equilibrated 5-ml HisTrap HP nickel-Sepharose column (GE Healthcare), washed with buffer containing 25 mM Tris-HCl, pH 7.9, 500 mM NaCl, 30 mM imidazole followed by a wash with buffer containing 25 mM Tris-HCl, pH 7.9, 500 mM NaCl, 60 mM imidazole, an overnight wash with buffer containing 25 mM Tris-HCl, pH 7.9, 500 mM NaCl, 0.1 % Triton X-114, followed by a wash with buffer containing 25 mM Tris-HCl, pH 7.9, 500 mM NaCl. EBD-200 was eluted with buffer containing 25 mM Tris-HCl, pH 7.5, 500 mM NaCl, 500 mM imidazole, 100 mM glycine, 2 mM TCEP, and 1 mM beta mercaptoethanol (BME). The His-SUMO tag was cleaved by dialysis with SUMO protease. The protein was concentrated and loaded onto a Superdex 200 Hi Load 26/60 gel filtration column (GE Healthcare) pre-equilibrated with 25 mM Tris-HCl, pH 7.5, 190 mM NaCl, 50 mM glycine, 2.5 mM BME. The protein eluted as a single peak at the expected molecular weight of the tetramer, and the fractions were pooled, concentrated, and frozen at −80 °C.

### 2.3. Expression and purification of the WT and Q59L mutant of the E. coli L-ASNase

WT and Q59L mutant of the *E. coli* L-ASNase, in a His6-SUMO-pET14b vector, were overexpressed in C41(DE3) cells. Induction with 0.3 mM IPTG was done when cell density reached an OD600 of 0.6–0.8 and then grown at 18 °C overnight. Cells were lysed by sonication and after centrifugation, the supernatant was loaded on a 5 ml Cytiva HisTRAP HP Ni^+^ column. After washing the column with ~300 mL wash buffer 1 (25 mM Tris pH 7.9, 500 mM NaCl, 30 mM imidazole) then ~200 mL wash buffer 2 (25 mM Tris pH 7.9, 500 mM NaCl, 60 mM imidazole) at a rate of 5 mL/min, the proteins were eluted using 25 mM Tris pH 7.9, 500 mM NaCl, 500 mM imidazole. The eluted His-SUMO fusion proteins were then cleaved using SUMO protease while dialyzing in buffer 1, and then the cut protein mixture was flowed back onto the Ni column to bind any uncleaved protein and the SUMO protease. The Ni column flowthrough was concentrated and then loaded onto a HiLoad 26/60 Superdex 200 size exclusion column. Fractions corresponding to the WT and Q59L proteins were combined, flash frozen in liquid nitrogen, and kept at −80 °C.

### 2.4. L-ASNase and L-GLNase activity assays

The catalytic activity of EBD-200 was determined using continuous spectroscopic enzyme-coupled assays to measure the generation of product through the 1:1 oxidation of reduced NADH. The conversion of NADH to NAD+ was measured spectrophotometrically as a decrease in absorbance at 340 nm at 37 °C. Asparaginase activity was measured using a modified assay [53] whereby L-aspartate generated by the hydrolysis of L-ASN (Sigma-Aldrich A93003) was converted using ~0.1 IU malic dehydrogenase (Sigma M2634) and ~10 IU glutamic-oxalacetic transaminase (Sigma G2751) helper enzymes. For the glutaminase assay, hydrolysis of L-GLN (ThermoFisher 25030149) was measured through conversion of the generated ammonia by ~5 IU L-glutamic dehydrogenase (Sigma G2626) helper enzyme. The kinetic assay buffer contained 50 mM Tris-HCl pH 7.5, 200 mM NaCl, 3.1 mM α-ketoglutarate, and 200 μM NADH. L-ASN and L-GLN stocks were made fresh using buffer containing 25 mM Tris pH 7.5 and 155 mM NaCl. Reactions were triggered using enzyme after equilibration with the substrate. Measurements were conducted in duplicate.

### 2.5. MS analysis of WT and Q59L E. coli L-ASNase preparations

MS analysis was performed by the UIC Research Resources Center Mass Spectrometry Core.

#### Sample Preparation:

The purified protein samples were digested using in-solution tryptic digestion protocol. In brief, the protein was reduced by incubation at 56 °C for 30 min at a final concentration of 20 mM Dithiothreitol, followed by the alkylation with IAA at a final concentration of 50 mM IAA for 20 min in darkness. The proteins were digested using Trypsin (enzyme/protein ratio at 1: 50) in 50 μL 0.1 M ammonium bicarbonate buffer at 37 °C for overnight. The digested peptides were desalted using C18 zip-tip and dried and re-suspended in 5 % acetonitrile, 0.1 % formic acid buffer for LC-MS analysis.

#### Instrumentation:

The digested peptides were analyzed using Q Exactive HF mass spectrometer coupled with an UltiMate 3000 RSLC nanosystem with a Nanospray Frex Ion Source (Thermo Fisher Scientific). Digested peptides were loaded into a Waters nanoEase M/Z C18 (100 Å, 5 μm, 180 μm × 20 cm) trap column and then separated by a 75 μm × 150 mm Waters BEH C18 (130 Å, 1.7 μm, 75 μm × 15 cm) at a flow rate of 300 nL/min. Solvent A was 0.1 % FA in water and solvent B was 0.1 % FA, 80 % ACN in water. The solvent gradient of LC was 5 % B in 3 min, 10 % B in 3 min, 10–35 % B in 30 min, 35–95 % B in 3 min, wash 95 % in 5 min, followed by 5 % B equilibration.

Full MS scans were acquired over 370–1400 *m/z* range with a resolution of 120,000 (at 200 *m/z*). The AGC target value was 3.00E+06 for the full scan. The 15 most intense peaks with charge states 2, 3, 4, 5 were fragmented in the HCD collision cell with a normalized collision energy of 28 %. The dynamic exclusion window was set to 30s. The tandem mass spectrum was acquired in the mass analyzer with a resolution of 60,000. The AGC target value was 1.00E+05. The ion selection threshold was 1.00E+04 counts, and the maximum allowed ion injection time was 50 ms for full scans and 50 ms for fragment ion scans.

#### Data Analysis:

Spectra were searched against the modified FASTA database with the WT and Q59L proteins using MaXQuant (version 2.0.3.0) with the following parameters; parent mass tolerance of 10 ppm, constant medication on cysteine alkylation, variable modification on methionine oxidation, deamidation of asparagine and glutamine. Search results were imported into Scaffold Q + S software (v5.1.2, Proteome Software, Portland, OR) for compilation, normalization, and comparison of spectral counts, etc. The filtering criteria of protein identification were a 1 % false discovery rate (FDR) of protein and peptide with 2 minimum peptide count.

The ratio of WT and Q59L species in each sample was calculated by the ratio of the unique peptide of P1 (GEQVVNIGSQDNDNVWLTLAK for WT) and P2 (GEQVVNIGSLDNDNVWLTLAK for Q59L). The intensity of P1 or P2 were normalized by the sum of the intensity of all peptides detected in the sample. Then the normalized values were used to represent the ratio of the corresponding protein species. P1 and P2 were confidently identified by MS/MS via HCD mode (with low error of precursor ions and high b and y series of ion coverage). See Fig. S2.

### 2.6. Pharmacokinetics study

For the pharmacokinetics study, healthy wild-type C57BL/6J male mice (10 weeks; #SC-C57J-F Janvier labs, RRID:IMSR_JAX:000664) were intraperitoneally injected with 3000 IU/kg EBD-200. For the activity determinations, 50 μL of peripheral blood was collected at regular timepoints (0, 4, 8, 24, 48, 72, 96, 168 and 216 h) and samples were processed according to the protocol described above.

### 2.7. Repeated dose toxicity study

Healthy wild-type C57BL/6J (#SC-C57J-F Janvier labs, RRID: IMSR_JAX:000664) mice (10 weeks old; n = 21) received repeated doses of either PEG-ASNase (3000 IU/kg; Oncaspar^®^ from Servier), EBD-200 (3000 IU/kg) or vehicle buffer control (25 mM Tris, 190 mM NaCl, 50 mM Glycine, 2 mM BME; pH 7.5). PEG-ASNase mice (n = 7) received 1 dose every 7 days (3 administration doses in total), while EBD-200 mice (n = 7) received 1 dose every 4 days (5 administration doses in total). 24 h after the last L-ASNase shot, blood was collected and hematologic (Vetscan^®^ HM5 Hematology Analyzer; Abaxis) and biochemical analyses (VetScan^®^ VS2 Chemistry Analyzer; Abaxis) were performed. For detailed methodology, see the Supplementary of our previous paper [31].

### 2.8. In vitro cell viability assays

The melanoma A2058 parental and A2058 *ASNS*-KO lines were the kind gift of Dr. Giorgio Galli. The liver cancer cell lines were obtained from the JCRB Cell Bank; JHH2 (RRID:CVCL_2786), JHH4 (RRID: CVCL_2787), JHH5-luc (RRID:CVCL_JF88), JHH6 (RRID:CVCL_2788) and JHH7 (RRID:CVCL_2805). The melanoma and liver cells were cultured in RPMI-1640 medium supplemented with 10 % FCS, L-glutamine (2 mM), and Penicillin/Streptomycin. All cell lines were analyzed by STR (short tandem repeat) and confirmed to match 100 % to corresponding STR profile data from the Global Bioresource Center ATCC. All cell lines were verified to be mycoplasma free. For determining cell viability-proliferation, the Alamar Blue (Invitrogen) assay was used as previously described [51].

### 2.9. In vivo treatment of B-ALL xenograft model with EBD-200

NSG-SGM3 (#013062, The Jackson Laboratory, RRID: IMSR_JAX:013062) mice (n = 18) were intravenously injected at 10 weeks of age with 4.5 × 10^6^ luciferase-positive SUP-B15 cells (a gift from Dr. Michael Jensen and previously used by us [51]). At regular time points, engraftment of these SUP-B15 cells was measured via bioluminescent imaging (BLI) using the IVIS Lumina II imaging system (PerkinElmer). After evidence of leukemic cell engraftment, mice were randomly equally divided into a vehicle buffer (25 mM Tris, 190 mM NaCl, 50 mM glycine, 2 mM BME; pH 7.5), PEG-ASNase and EBD-200 treatment group. EBD-200 was administered via intraperitoneal injection at a dose of 3000 IU/kg twice a week (total of 10 administration doses) and PEG-ASNase at a dose of 1500 IU/kg once a week (total of 5 administration doses). The lower dose and frequency of Oncaspar treatment was done to account for its longer *in vivo* half-life compared to EBD-200. During the experiment, leukemic burden was evaluated via BLI at regular timepoints, and mice were monitored daily for clinical signs of ALL disease or drug-induced toxicity. This experiment was performed according to the ethical guidelines of UGhent regulations, with approval of the ethical committee for laboratory animal experimentation of the Faculty of Medicine and Health Sciences.

### 2.10. In vivo therapy efficacy studies with EBD-200 in melanoma and liver cancer xenograft models

For the A2058 melanoma study, athymic nude mice (#490, Charles River Laboratories) were injected with 5 × 10^6^ A2058 parental (n = 11) or A2058 *ASNS* KO (n = 12) melanoma cells in the right flank. For the JHH5 liver study, 2 × 10^6^ JHH5 cells with 1:1 Matrigel were implanted in the right flank of athymic nude mice (CRL, strain 490; n = 12). When the volume of the tumors reached 100–350 mm^3^, mice were assigned to a vehicle buffer or EBD-200 treatment group (3000 IU/kg via intraperitoneal injection 2 times a week). The A2058 and JHH5 mice were sacrificed once tumor volumes >1000 mm^3^. These experiments were performed according to the ethical guidelines of The University of Illinois at Chicago, with approval of the ethical committee for laboratory animal experimentation.

## 3. Results and discussion

### 3.1. Correlation between the L-GLNase co-activity and toxicity

Different to the L-ASNase activity, which can achieve and maintain asparagine depletion, the L-GLNase co-activity of the *E. coli* L-ASNase seems to transiently perturb the high glutamine levels *in vivo* [54–57]. This perturbation of glutamine levels can potentially impact both the efficacy and tolerability of the drug. Earlier clinical studies implicated L-GLNase activity with toxicity. Specifically, clinical evaluation of the *Acinetobacter* glutaminase-asparaginase (AGA, an asparaginase with more pronounced L-GLNase activity compared to the *E. coli* L-ASNase) in adult leukemia patients resulted in severe central nervous system toxic effects, hyperglycemia, and respiratory alkalosis [58]. This was consistent with two earlier clinical studies with AGA in which the authors noted “nausea, vomiting, weight loss, transient fever, and hyperglycemia without ketosis or acidosis appear to be more frequent with the succinylated AGA preparations than with that reported with *Escherichia coli* asparaginase” [59], and “reversible depression of the central nervous system, ranging from encephalopathy to coma, occurred in a dose-related manner and was dose limiting. Other prominent reactions included respiratory alkalosis, hyperglycemia, nausea, and vomiting” [60]. Additional data correlating the L-GLNase activity and toxicity comes from studies that compared the treatment of mice with *E. coli* L-ASNase to that with the *Vibrio succinogenes* (now called *Wolinella succinogenes*) L-ASNase, which was reported to have low L-GLNase co-activity. These studies found that the “hepatotoxicity of *Escherichia coli* asparaginase parallels the toxicity observed in humans with a rapid increase in liver lipid levels and decreased plasma levels of albumin, antithrombin III, cholesterol.” In contrast, the *W. succinogenes* L-ASNase was not associated with significant hepatotoxicity [55,61–63]. Other studies associated the L-GLNase activity with perturbations in blood coagulation [54] and in reducing protein synthesis in the liver [64]. Together, these studies suggest a direct correlation with the level of the L-GLNase activity and toxicity.

Our studies have also shown a strong correlation between L-GLNase co-activity and increased toxicity. In one study we compared Erwinaze (*Erwinia chrysanthemi* L-ASNase; *Er*A) to an engineered *Er*A triple mutant (*Er*A-TM) that has fully preserved the L-ASNase activity but is mostly devoid of L-GLNase co-activity. We observed significantly reduced toxicity for *Er*A-TM compared to *Er*A [51]. Increased tolerability of EBD-200 over PEG-ASNase, presented below, also demonstrates this correlation. Of note, at this point it is unclear whether the observed direct correlation between toxicity and level of L-GLNase co-activity is a result of transient reduction in L-glutamine levels or the accumulation of glutamate and/or ammonium and other downstream effects.

### 3.2. Refuting the published claims for the requirement of L-GLNase co-activity for L-ASNase efficacy

While the data correlating increased toxicity of L-ASNases with L-GLNase co-activity is strong, the contribution of the L-GLNase co-activity to its anti-cancer efficacy is still highly debated [15,31, 65–68]. We believe that part of the controversy regarding the role of the L-GLNase activity is due to conclusions based solely on cell culture data [67–69]. Different to an *in vivo* study, in a dish one achieves complete and sustained glutamine depletion with the bacterial L-ASNases. Since *in vitro* most cells lines require glutamine as a supplemental media component for survival, it is of no surprise that an L-ASNase with a L-GLNase co-activity shows higher efficacy (i.e. lower IC_50_ values) as an artefact when compared to a L-ASNase that lacks such a co-activity.

Also contributing to the controversy regarding the role of the L-GLNase activity is the use of L-ASNase with Km properties that are too high for achieving sustained asparagine depletion. In work executed by several independent laboratories using our engineered L-ASNase variants, strong *in vivo* therapy efficacy was observed with L-ASNase that either had negligible [50] (*ErA-TM*), or zero (EBD-200; this study) L-GLNase activity. Therefore, we questioned several reports that claimed an essential role of the L-GLNase co-activity in vivo [56,70]. One such report employed the Q59L mutant of the *E. coli* L-ASNase, which claimed to be L-GLNase-free compared to the wild-type enzyme [70,71]. One possible explanation for this discrepancy relates to the L-ASNase Km value of the Q59L mutant. To achieve complete depletion of asparagine, the Km for this amino acid must be in the low micromolar range (which is why one cannot use a human L-ASNase, with its >3 mM km and physiologic concentration of ASN in blood being ~50 μM). Since the Km value of the Q59L was not reported in the article or associated patent application, we generated this mutant ourselves and characterized its L-ASNase kinetic properties. Compared to the wild type *E. coli* L-ASNase, we observed that the Q59L mutation reduced the rate of the L-ASNase reaction by 97 % (from 44 to 1.3 sec^−1^) and most importantly, increased the Km 100-fold, from 14 μM to 1.4 mM (Fig. S1A).

Such changes to its kinetic properties essentially render this enzyme ineffective as an L-ASNase, and therefore would not be expected to show any meaningful efficacy, especially *in vivo*. Yet, several reports observed *in vivo* efficacy [56,70]. To understand this puzzle, we gratefully received a sample of the *E. coli* Q59L enzyme from its developer [71]. When we examined its kinetics properties, we were surprised to measure a Km of 320 μM. While this Km value is still too high for achieving sustained asparagine depletion *in vivo*, it is just low enough that at high concentrations one would observe an effect on the proliferation of ASNS-negative cells *in vitro*. We conclude that the reported reduced *in vivo* durable therapeutic potential of Q59L is most likely not a result of its lack of L-GLNase co-activity as was previously concluded, but rather from the perturbation of the functionality of its L-ASNase activity.

This still left the question as to why the Q59L protein produced in our laboratory showed a Km > 1.4 mM, and the gifted Q59L protein showed a Km of 320 μM. We made the hypothesis that the gifted Q59L is a mixture of WT (that has a Km of 15 μM) and Q59L (that has a Km of >1.4 mM). Such a mixture would be impossible to detect on an SDS-PAGE gel (the MW is nearly identical) (Fig. S1B). Moreover, since this family of L-ASNases arrange into a tetramer, it is possible that the gifted Q59L formed hetero-tetramers containing both WT and Q59L protomers. Importantly, when expressing our versions of WT and Q59L *E. coli* L-ASNases in *E. coli*, we bypassed this potential complication by avoiding the natural periplasmic processing of this enzyme keeping ours sequestered in the cytosol away from the WT enzyme and by also adding a large tag to our Q59L mutant that allowed us to verify using SDS-PAGE the absence of any WT *E. coli* L-ASNase contamination. In contrast, the gifted Q59L was expressed with a pelB leader sequence that would traffic the protein through the periplasm, making it available to associate with endogenous *E. coli* L-ASNase. To test the hypothesis that the gifted Q59L enzyme is a mixture of WT and Q59L, we evaluated by mass spectrometry the gifted WT and Q59L proteins and the corresponding WT and Q59L proteins made by us. As expected, both the gifted and our WT *E. coli* preparations were observed to be consistent with 100 % WT *E. coli* L-ASNase. In contrast, the gifted Q59L was observed to in fact only consist of a minority 25 % Q59L, with the remaining majority 75 % being the WT enzyme. For the Q59L produced by us in house, the amount of contaminating WT sequence was estimated at a negligible 0.6 % (Fig. S2).

We reiterate the critical importance of a low Km value of L-ASNase for achieving complete and sustained asparagine depletion *in vivo*. The FDA-approved bacterial L-ASNases have a Km value of 15–50 μM, and we conclude that the reported observed lack of efficacy of L-ASNases that lack L-GLNase co-activity is directly a result of their Km being too high for achieving complete and sustained asparagine depletion *in vivo* (but low enough to demonstrate some effect, especially *in vitro*). In sum, our results challenge the conclusion that L-GLNase activity is required for L-ASNase efficacy against ASNS^low^ cancer cells. Since our earlier work demonstrated that L-GLNase co-activity contributes to the side effects of L-ASNase treatment [31,51], we made the prediction that an L-GLNase-free L-ASNase would possess equivalent efficacy with reduced toxicity. To test this further, we developed and evaluated a novel L-ASNase that we refer to as EBD-200.

### 3.3. Development of EBD-200: a human-like L-ASNase with clinically relevant enzymatic properties and high specificity for asparagine

Unlike the *E. coli* and *Erwinia* L-ASNases, which possess glutaminase co-activity (2 % and 10 % relative to their L-ASNase activity, respectively), the guinea pig L-ASNase (*Gp*A; Gene name ASPG; Uniprot ID H0W0T5) is highly specific for asparagine [52]. An additional difference between the bacterial asparaginases and their mammalian homologs is the presence of a C-terminal domain that follows the N-terminal asparaginase domain, which is composed of several ankyrin repeats. For the human homolog (*h*A; Gene name ASPG; Uniprot ID Q86U10), this C-terminal domain (which is highly similar to the C-terminal domain of *Gp*A) has been annotated to exhibit several enzymatic properties such as lysophospholipase, transacylase, and PAF acetylhydrolase). Since our intention is to use *Gp*A as a therapeutic, we want to avoid the introduction of potentially toxic activities from the C-terminal domain. Therefore, we sought the minimal domain that contained only the asparaginase activity. In our previous work [52], we observed that the 565-residue full-length *Gp*A got cleaved at residue ~359 during the crystallization experiment, which was the last residue we could model in the crystal structure. Therefore, we expressed several *Gp*A truncation variants that end at residue 359 or beyond and discovered that a deletion construct spanning residues 1–369 expressed as a stable protein with L-ASNase activity – referred to here as *Gp*A369.

**Fig. 1.**
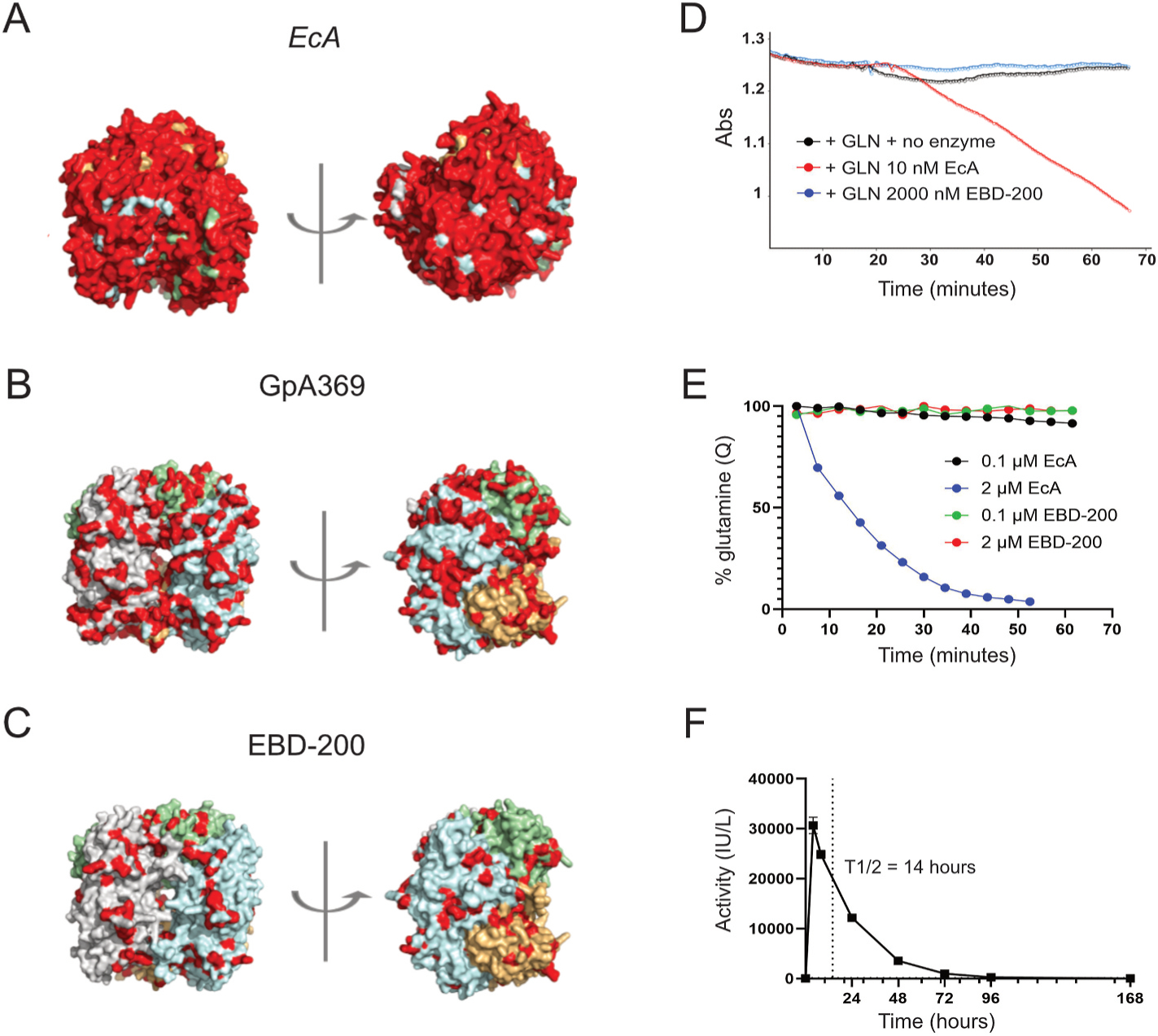
EBD-200 presents a surface more similar to that presented by hA and has optimal in vivo asparaginase kinetic properties with no L-GLNase co-activity resulting in an improved toxicity profile compared to PEG-ASNase EBD-200 presents a surface that is significantly more identical to the surface of the human asparaginase homolog compared to the E. coli asparaginase (EcA) or the truncated wild type guinea pig asparaginase (GpA369). Shown are surface representations of the tetrameric enzymes. (A) EcA;(B) GpA369; (C) EBD-200. Each protomer in the tetramer is shown in a different color (gray, green, blue or orange), except for residues that are different to the human enzyme, which are shown in red. (D) L-GLNase activity measurements in a continuous enzyme-coupled spectrophotometric assay using an excess of 5 mM GLN and triggered with either no enzyme (black trace) as a negative control, 10 nM EcA (red trace), or 2000 nM EBD-200 (blue trace) where GLNase activity is easily observed by EcA but not by EBD-200 whose activity is the same as the negative control. EB) L-GLNase activity as measured by NMR spectroscopy using an excess of 5 mM GLN whereby EcA demonstrates strong L-glutaminase activity at a concentration of 2 μM (red trace) and residual activity at 0.1 μM (black trace) whereas at both 0.1 μM (green trace) and 2 μM (blue trace), EBD-200 demonstrates a clear lack of GLNase activity. (F) L-ASNase activity measurements in blood plasma samples of mice obtained at 4, 8, 24, 48, 72, 96 and 168 h after administration of 3000 IU/kg of EBD-200 show that EBD-200 activity is still above the therapeutic threshold of 100 IU/L 96 h after injection. Based on this pharmacokinetic profile, the EBD-200 treatment regimen was determined. Mean with SEM is plotted. Molecular models of EcA, GpA369 and EBD-200 were generated using the AlphaFold Server. (For interpretation of the references to color in this figure legend, the reader is referred to the Web version of this article.)

The observed immunogenicity of the *E. coli* asparaginase is not surprising, having only 26.9 % amino acid identity to *h*A, which results in a surface that mostly presents residues different to those presented by *h*A (Fig. 1A). *Gp*A369 has much higher sequence identity to *h*A (72.2 %) resulting in a surface more similar to that of *h*A (Fig. 1B). In order to reduce the immunological risk of *Gp*A369 further, we commenced a process of humanization. We mutated select residues in *Gp*A369 to their corresponding type as present in *h*A, generating EBD-200. First, we exploited our crystal structure to guide the selection of residues that were predicted not to be detrimental to the L-ASNase activity when mutated. Second, we performed a T cell proliferation assay to identify the presence of potential T cell epitopes in our clone. The identified sites were then mutated to the corresponding sequence in *h*A. Together, this process increased the amino acid identity to *h*A from 72.2 % to 83.0 % (Table S1), yielding EBD-200. EBD-200 presents a surface that is even more similar to *h*A (Fig. 1C). We are aware that while this humanization process may not fully eliminate the immunological risk, it is predicted to reduce it due to tolerance. Importantly, we verified that the humanizing mutations did not negatively impact the favorable L-ASNase kinetics properties as present in *Gp*A and that the high specificity for asparagine was maintained in EBD-200 (Table 1; Fig. 1D and E).

Besides evading neutralization by the immune system and having a low Km for asparagine that is comparable to that of the FDA-approved bacterial L-ASNases, an additional property that influences the therapeutic efficacy of L-ASNases is *in vivo* persistence. The FDA-approved, naked bacterial L-ASNases have a very short half-life – in mice between 2.3 and 3.5 h. In contrast, EBD-200 has a half-life in mice of 14 h (Fig. 1F), allowing for less frequent administrations to maintain asparagine depletion.

Prior to evaluating the therapeutic efficacy of EBD-200, we performed a 2-week repeat dosing toxicity experiment in wild-type mice. Similar to our previously reported *Er*A-TM [31] with ultra-low glutaminase co-activity, we anticipated that this novel humanized *Gp*A derived L-ASNase with no intrinsic L-GLNase activity would also have an improved toxicity profile compared to the PEG-ASNase. Results show that EBD-200, unlike PEG-ASNAse, has no significant effect on albumin, total protein and blood platelets. However, EBD-200 and PEG-ASNase do show a comparable decrease in white blood cell (WBC) and lymphocyte count which suggests that L-GLNase co-activity might not be involved in L-ASNase observed toxicity in WBCs and lymphocytes (Fig. S3). Endothelial damage and coagulation problems are often seen in patients treated with bacterial ASNases, but in this study, possibly due to the small number of animals and the single dose study design, we did not observe these toxicities in the EBD-200 or PEG-ASNase treated animals.

### 3.4. EBD-200 has a significant anti-leukemic effect on SUP-B15 cells in vivo

**Fig. 2.**
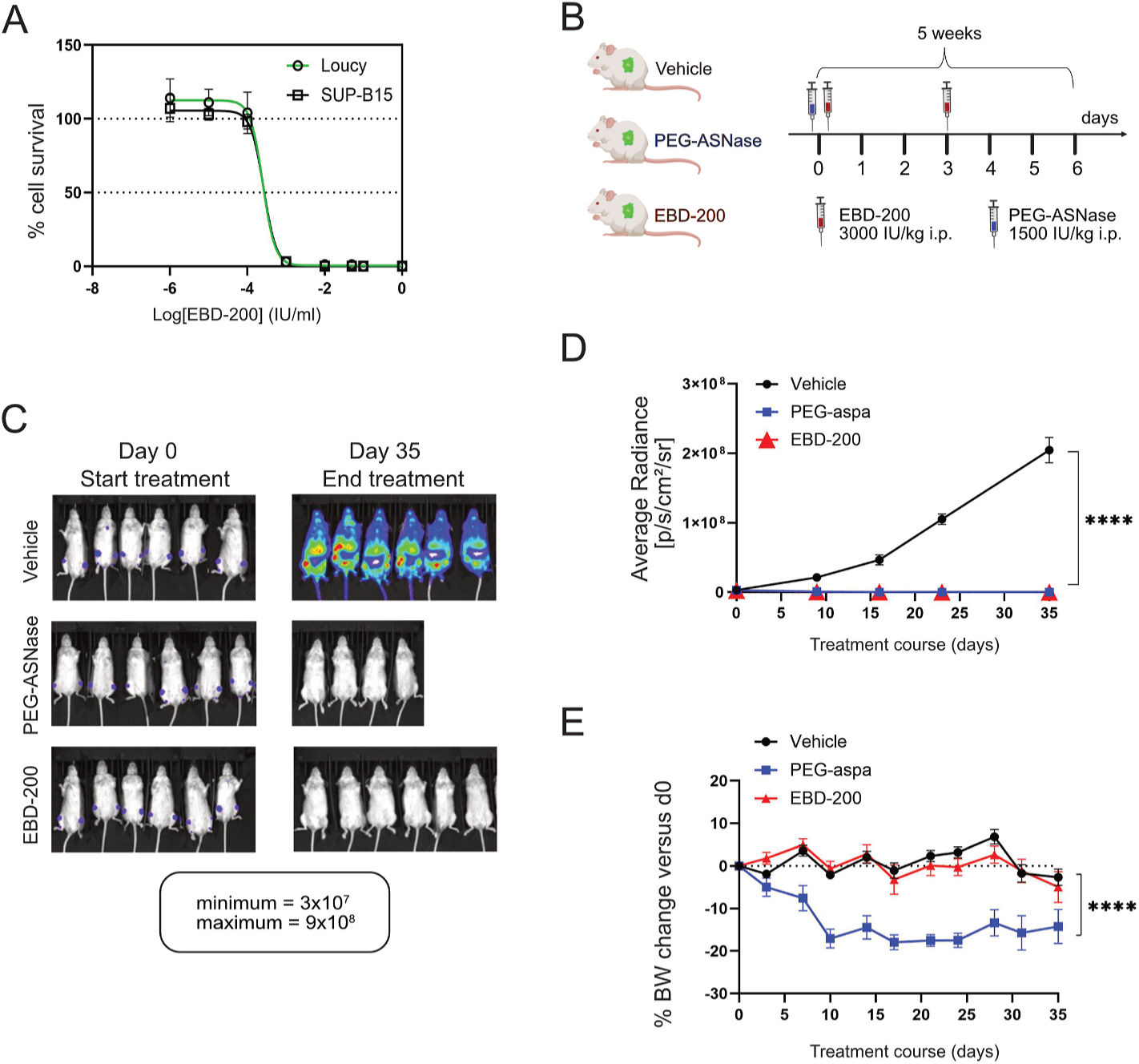
EBD-200 has a significant anti-leukemic effect on SUP-B15 cells in vivo. **(A)** The ALL cell lines SUP-B15 and LOUCY show a clear *in vitro* sensitivity to EBD-200 with an EC_50_ of 0.62 and 0.34 mIU/ml respectively. Mean with SEM is plotted. **(B)** To test the *in vivo* anti-leukemic potential of EBD-200, mice were injected with luciferase + SUP-B15 cells and treated for 5 weeks with either vehicle buffer, PEG-asparaginase (once a week; 1500 IU/kg i.p.) or EBD-200 (two times a week; 3000 IU/kg i.p.). Treatment schedule was based on the previous determined pharmacokinetic profile of EBD-200 **(C) and (D)** Leukemic burden was monitored via BLI imaging during the L-ASNase treatment regimen. Results show a steady increase in leukemic cells in the vehicle group, while no leukemia signal could be observed in the PEG-asparaginase and EBD-200 treated mice after 5 weeks of L-ASNase therapy. This clearly demonstrates that glutaminase co-activity is not required to kill off ALL cells. For panel D, the mean with SEM is plotted (p < 0.0001; Mixed Effects Model). **(E)** Body weights of the mice were carefully monitored during treatment and shows that PEG L-ASNase treated mice lose significantly more weight than EBD-200 treated mice (p < 0.0001; Mixed Effects Model). In general, our results show that the glutaminase-free EBD-200 has a similar anti-leukemic effect as PEG-asparaginase in SUP-B15 but with significantly less co-glutaminase induced toxic side effects. Mean with SEM is plotted.

As ALL cells are known to be highly sensitive towards L-ASNase treatment, ALL models serve as an ideal model to evaluate the anti-cancer therapeutic potential of alternative L-ASNase variants, including our novel EBD-200. First, we confirmed the *in vitro* sensitivity to EBD-200 for LOUCY (T-ALL) (EC_50_ = 0.62 mIU/ml) and SUP-B15 (B-ALL) (EC_50_ = 0.34 mIU/ml), two ALL cell lines previously reported to be sensitive to L-ASNase therapy (Fig. 2A). Next, we tested the *in vivo* efficacy of asparagine deprivation using the L-GLNase-free EBD-200 in a luciferase positive SUP-B15 xenograft mouse model. Following successful engraftment and upon evidence of leukemia progression, mice were randomized in three groups (6 mice/group) based on *in vivo* bioluminescence imaging (BLI) signals and treated with either vehicle buffer, PEG-ASNase or EBD-200 to maintain continuous asparagine deprivation in peripheral blood for 5 weeks (Fig. 2B). Leukemia burden and progression were monitored at regular timepoints via BLI measurement. Mice in the control group, treated with the vehicle buffer only, succumbed to leukemia progression at the end of the 5-week treatment period. In contrast, the leukemic mice treated with PEG-ASNase and EBD-200 show no signs of ALL disease progression at the end of the 5-week treatment (Fig. 2C and D). Similar to our previous study [31], these results unrebuttably demonstrate that L-GLNase co-activity is not essential for the anti-leukemic properties of L-ASNases. Notably, during the treatment protocol, PEG-ASNase treated mice lost significantly more body weight than the EBD-200 treated mice (p < 0.0001; Mixed Effects Model) and two mice needed to be sacrificed after the second dose of PEG-ASNase administration as they reached the pre-set humane endpoints of our study (Fig. 2E). These results convincingly show that L-GLNase-free EBD-200 has comparable leukemic inhibitory activity to PEG-ASNase, but with significantly less toxicity. This experiment was performed according to the ethical guidelines of UGhent regulations, with approval of the ethical committee for laboratory animal experimentation of the Faculty of Medicine and Health Sciences.

### 3.5. EBD-200 effectively kills ASNS-KO melanoma cells in vivo

**Fig. 3.**
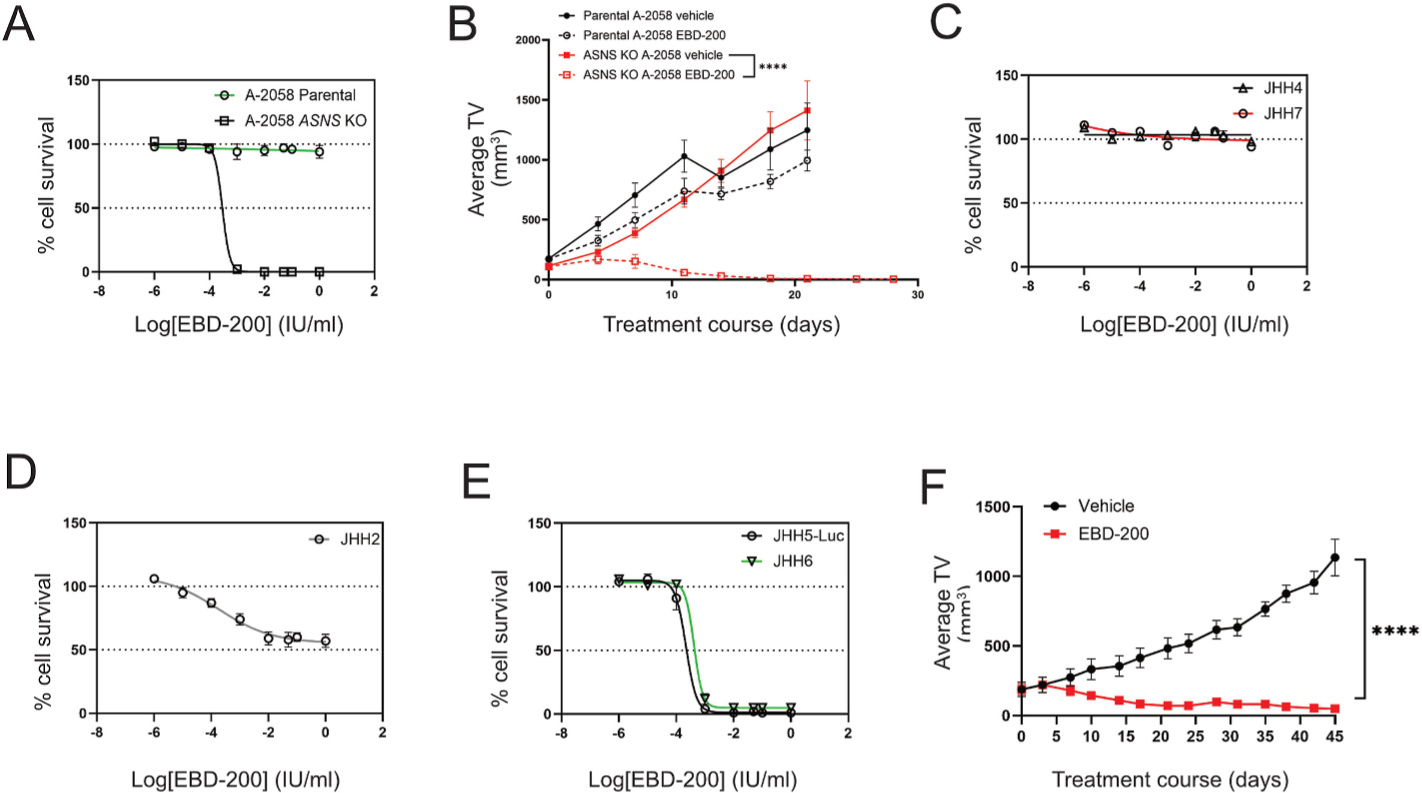
EBD-200 *effectively kills ASNS KO melanoma and JHH5 liver cells in vivo. (A) A2058 parental cells (ASNS expressing) were not shown to be sensitive to EBD-200 in vitro while A2058 ASNS KO clearly show EBD-200 sensitivity (EC*_*50*_ = *0.3 mIU/ml). This shows that ASNS expression can be considered a marker for L-ASNase sensitivity in this A2058 cell line model.* ***(B)*** *The therapeutic potential of EBD-200 was evaluated in A2058 parental and A2058 ASNS KO xenograft mouse models. While EBD-200 has no effect on the ASNS-expressing A2058 parental cells, highly potent tumor cell killing is observed as a significant reduction in average tumor volume (TV) in the EBD-200 treated A2058 ASNS KO cells (p* < *0.0001; Mixed Effect Model).* ***(C-E)*** *Our in vitro screening on five HCC cell lines shows that JHH4 and JHH7 are not sensitive towards EBD-200, while JHH5-luc (EC*_*50*_= *0.26 mIU/ml) and JHH6 (EC*_*50*_= *0.74 mIU/ml) show a clear EBD-200 sensitivity. For the JHH2 cell line (EC*_*50*_= *0.63 mIU/ml) we observed only partial sensitivity demonstrated by a reduction by only 50 % in cell survival, which likely due to the upregulation of ASNS in this cell line.* ***(F)*** *A JHH5-luc mouse xenograft model was established and once tumors reached 100 mm*^*3*^*, EBD-200 or vehicle buffer treatment was initiated. At day 45, a significant decrease in JHH5-luc tumor burden between EBD-200 and vehicle treated mice was observed (p* < *0.0001; Mixed Effect Model). For all panels, the mean with SEM is plotted*.

Our lead hypothesis is that cancer cells that (i) do not express ASNS and (ii) cannot upregulate ASNS expression in response to asparagine depletion will be sensitive to EBD-200. As such, we were surprised to read a 2021 report in which melanoma cells that were deleted of ASNS by CRISPR did not show sensitivity to L-ASNase *in vivo*, while showing sensitivity *in vitro* [72]. However, upon close inspection of the *E. coli* L-ASNase dose that was used in these reported experiments (2000 IU/kg, 3 times/week) and lack of evidence of sustained asparagine depletion, we speculated that the reason for the lack of *in vivo* efficacy was due to suboptimal dosing and frequency that was unable to maintain complete asparagine depletion, which allowed the melanoma ASNS-KO cells to recover and prevented tumor cell death. Note that using a higher dose or more frequent dosing of the *E. coli* asparaginase in mice would result in severe weight loss that triggers the humane endpoints. Since our EBD-200 allows for a dosing regimen that maintains high L-ASNase activity between treatments without causing unacceptable weight loss, we predicted that the ASNS-KO melanoma cells would respond to EBD-200 *in vivo*. To test this prediction, we obtained the exact same A2058 parental and ASNS-KO melanoma cells from Apfel et al. [72] and repeated their experiments with our L-GLNase-free EBD-200. Our *in vitro* results confirm that ASNS-depleted A2058 melanoma cells are sensitive towards EBD-200, while the parental ASNS-positive A2058 cell line does not show a L-ASNase response (Fig. 3A). For the *in vivo* experiment, we used the same dose of 3000 IU/kg twice/week as was used in the SUPB15 ALL study described above. As expected, based on our hypothesis, tumor growth of the parental ASNS-positive A2058 melanoma cells (i.e. those with ASNS) was unhindered by EBD-200 treatment. In contrast, tumors formed by the ASNS-null A2058 melanoma cells rapidly regressed in response to EBD-200 (Fig. 3B), directly contradicting the results from Apfel et al. This result supports our model that cells lacking ASNS can be eliminated by our L-GLNase-free L-ASNase. Importantly, combined with the improved tolerability due to the lack of L-GLNase co-activity as demonstrated by bodyweight loss during treatment (Fig. S4), this suggests the EBD-200 could be effective against any ASNS^low^ tumors.

### 3.6. HCC cells that possess a hypermethylated ASNS promoter are sensitive towards EBD-200 in vivo

The ability of amino acid deprived cancer cells to upregulate ASNS expression and resist ASNase therapy may depend on the ASNS promoter methylation status. It is hypothesized that hypermethylation of the ASNS promotor prevents ATF4 to bind and reactivate the promotor. A hallmark report in 2019 searching for metabolic vulnerabilities in >900 human cancer cell lines discovered that a subset of stomach and liver cancers share the L-ASNase sensitivity signature of ALL cells (low ASNS expression + hypermethylated ASNS promoter) [73]. For both stomach and hepatocellular carcinoma (HCC) cell lines containing this signature, *in vitro* sensitivity to *E. coli* L-ASNase with L-GLNase co-activity was reported, as well as *in vivo* sensitivity to one of the stomach cell lines.

Using EBD-200, we first confirmed the ASNase response/lack thereof for 5 HCC cell lines (JHH2, JHH4, JHH5-luc, JHH6 and JHH7). We observed a similar correlation between the previously reported level of ASNS promoter methylation and sensitivity (hyper being sensitive, hypo being resistant) to EBD-200 L-ASNase (Fig. 3C–E). Notably, this demonstrates that the L-GLNase co-activity present in the *E. coli* L-ASNase is not required for killing the hyper-methylated JHH5 and JHH6 cells *in vitro*. Next, we evaluated the *in vivo* EBD-200 response in the L-ASNase sensitive JHH5 liver model. By day 45 of treatment, the average tumor volume of the buffer control group was >1100 mm^3^. In contrast, at day 45, the average tumor volume of the EBD-200 treated group was significantly lower (<45 mm^3^; p < 0.0001; Mixed Effect Model). This strong reduction in tumor volume demonstrated the *in vivo* efficacy of our L-GLNase-free EBD-200 against this HCC model (Fig. 3F). Importantly, we could dose the animals continuously for 45 days with an acceptable weight loss (Fig. S5). To be clear, our claim is not that the lack of the L-GLNase activity eliminates all of the toxicity of L-ASNase treatment. But rather, that the L-GLNase activity is (i) not required for efficacy against cancer cells lacking ASNS and (ii) the toxicity is reduced such that a dose that maintains consistent L-ASNase activity that assures depletion of asparagine can be maintained for an extended period of time.

## 4. Conclusions

Severe side effects of current L-ASNase preparations limit their clinical use. Side effects can originate from three sources: (i) immune response against the enzyme drug; (ii) on-target depletion of asparagine; and (iii) off-target perturbation of glutamine levels. The human-like property of EBD-200 suggests it would be less immunogenic compared to the bacterial L-ASNases. The L-GLNase-free EBD-200 demonstrated potent cell killing in multiple mouse cancer xenograft models, where the prerequisite for response is a lack of ASNS, whether achieved by hypermethylation of the ASNS promoter or through a genetic KO. We cannot rule out that L-GLNase co-activity may play an efficacy role in the treatment of ALL endowed with a significant basal or inducible ASNS activity. But since L-GLNase co-activity comes with increased side effect, for those cancer cells that have low basal ASNS expression but a hypo-methylated promoter, we suggest that a combination with a GCN2 inhibitor should be explored as a strategy to expand the sensitivity to L-ASNase, as was previously suggested by Nakamura et al. [74]. Moreover, the high tolerability of EBD-200 allowed us to treat the mice over an extended period, which is challenging with the bacterial L-ASNases. Therefore, these data support the further development of our human-like L-GLNase-free L-ASNase for clinical use in blood and solid tumors.

## Supplementary Material

Supplemental

Appendix A. Supplementary data

Supplementary data to this article can be found online at https://doi.org/10.1016/j.canlet.2024.217404.

## Figures and Tables

**Table 1:** Enzymatic and pharmacokinetic properties of EBD-200, naked and PEGylated *E. coli* L-ASNase.

L-ASNase	EBD-200 ^a^	Naked *E. coli* L-ASNase (Spectrila) ^b^	PEGylated *E. coli* L-ASNase (Oncaspar) ^c^

Km for asparagine	41.3 ± 0.7 μM	15.0 ± 0.5 μM	10–20 μM
k_cat_ for asparagine	61.7 ± 0.7 sec^−1^	44.4 ± 0.3 sec^−1^	58 - 88 sec^−1^
Km for glutamine	NA	1.38 ± 0.09 mM	^e^
k_cat_ for glutamine	NA	0.89 ± 0.01 sec^−1^	^e^
Glutaminase activity	Undetectable	2 %	^e^
	***In vivo* persistence in mice**		
Half-life in mice	14 h ^a^	3.2 ^a^ to 3.35 h ^d^	68 h ^a^

NA: Not applicable – no enzymatic activity with glutamine.

a This work.

b Nguyen et al., J Biol Chem. 2016 Aug 19; 291(34): 17664–17676 [50].

c Faschinger and Sessler Adv Ther (2019) 36:2106–2121 [75].

d Narta et al. Critical Reviews in Oncology/Hematology 61 (2007) 208–221 [76].

e We could not find data in the literature for these. However, given that the L-ASNase kinetic parameters between the naked and PEGylated *E. coli* enzyme are virtually identical, it stands to reason that their L-GLNase activity is also highly similar.

## References

[1] Egler RA, Ahuja SP, Matloub Y, L-asparaginase in the treatment of patients with acute lymphoblastic leukemia, J. Pharmacol. Pharmacother. 7 (2) (2016) 62–71. 27440950 10.4103/0976-500X.184769 PMC4936081

[2] Van Trimpont M, Peeters E, De Visser Y, , Novel insights on the use of L-asparaginase as an efficient and safe anti-cancer therapy, Cancers 14 (4) (2022). 10.3390/cancers14040902 PMC8870365 35205650

[3] Lubkowski J, Vanegas J, Chan WK, , Mechanism of catalysis by l-asparaginase, Biochemistry 59 (20) (2020) 1927–1945. 32364696 10.1021/acs.biochem.0c00116

[4] Chiu M, Taurino G, Bianchi MG, Kilberg MS, Bussolati O, Asparagine synthetase in cancer: beyond acute lymphoblastic leukemia, Front. Oncol. 9 (2019) 1480. 31998641 10.3389/fonc.2019.01480 PMC6962308

[5] Lomelino CL, Andring JT, McKenna R, Kilberg MS, Asparagine synthetase: function, structure, and role in disease, J. Biol. Chem. 292 (49) (2017) 19952–19958. 29084849 10.1074/jbc.R117.819060 PMC5723983

[6] Cecconello DK, Magalhães MRd, Werlang ICR, Lee MLdM., Michalowski MB, Daudt LE, Asparaginase: an old drug with new questions, Hematol. Transf. Cell Therapy 42 (3) (2020) 275–282. 10.1016/j.htct.2019.07.010 PMC7417439 31801703

[7] Medawar CV, Mosegui GBG, Vianna CMM, Costa TMAD, PEG-asparaginase and native Escherichia coli L-asparaginase in acute lymphoblastic leukemia in children and adolescents: a systematic review, Hematol Transfus. Cell Ther. 42 (1) (2020) 54–61. 31412986 10.1016/j.htct.2019.01.013 PMC7031090

[8] Maese L, Rau RE, Current use of asparaginase in acute lymphoblastic leukemia/lymphoblastic lymphoma, Front Pediatr 10 (2022) 902117. 35844739 10.3389/fped.2022.902117 PMC9279693

[9] Maese L, Rizzari C, Coleman R, Power A, van der Sluis I, Rau RE, Can recombinant technology address asparaginase Erwinia chrysanthemi shortages? Pediatr. Blood Cancer 68 (10) (2021) e29169. 34105243 10.1002/pbc.29169

[10] Tong WH, Rizzari C, Back to the future: the amazing journey of the therapeutic anti-leukemia enzyme asparaginase, Haematologica 108 (10) (2023) 2606–2615. 37470157 10.3324/haematol.2022.282324 PMC10542841

[11] Asselin B, Rizzari C, Asparaginase pharmacokinetics and implications of therapeutic drug monitoring, Leuk. Lymphoma 56 (8) (2015) 2273–2280. 25586605 10.3109/10428194.2014.1003056 PMC4732456

[12] Gupta S, Wang C, Raetz EA, , Impact of asparaginase discontinuation on outcome in childhood acute lymphoblastic leukemia: a report from the children’s Oncology group, J. Clin. Oncol. 38 (17) (2020) 1897–1905. 32275469 10.1200/JCO.19.03024 PMC7280050

[13] Perusini MA, Andrews C, Eshetu AG, , Asparaginase completion among adults including older patients with acute lymphoblastic leukemia treated with a modified DFCI protocol, Leukemia 38 (4) (2024) 912–913. 38431747 10.1038/s41375-024-02201-1

[14] Pieters R, Hunger SP, Boos J, , L-asparaginase treatment in acute lymphoblastic leukemia: a focus on Erwinia asparaginase, Cancer 117 (2) (2011) 238–249. 20824725 10.1002/cncr.25489 PMC3000881

[15] Fonseca MHG, Fiúza TDS, Morais SB, Souza T, Trevizani R, Circumventing the side effects of L-asparaginase, Biomed. Pharmacother. 139 (2021) 111616. 33932739 10.1016/j.biopha.2021.111616

[16] Krishna M, Nadler SG, Immunogenicity to biotherapeutics - the role of anti-drug immune complexes, Front. Immunol. 7 (2016) 21. 26870037 10.3389/fimmu.2016.00021 PMC4735944

[17] Burke MJ, Zalewska-Szewczyk B, Hypersensitivity reactions to asparaginase therapy in acute lymphoblastic leukemia: immunology and clinical consequences, Future Oncol. 18 (10) (2022) 1285–1299. 35107320 10.2217/fon-2021-1288

[18] Veronese FM, Pasut G, PEGylation, successful approach to drug delivery, Drug Discov. Today 10 (21) (2005) 1451–1458. 16243265 10.1016/S1359-6446(05)03575-0

[19] Soares AL, Guimarães GM, Polakiewicz B, de Moraes Pitombo RN, Abrãhao-Neto J, Effects of polyethylene glycol attachment on physicochemical and biological stability of E. coli L-asparaginase, Int. J. Pharm. 237 (1–2) (2002) 163–170. 11955814 10.1016/s0378-5173(02)00046-7

[20] Gao Y, Joshi M, Zhao Z, Mitragotri S, PEGylated therapeutics in the clinic, Bioeng. Transl. Med. 9 (1) (2024) e10600. 38193121 10.1002/btm2.10600 PMC10771556

[21] Keating MJ, Holmes R, Lerner S, Ho DH, L-asparaginase and PEG asparaginase–past, present, and future, Leuk. Lymphoma 10 (Suppl) (1993) 153–157. 8481665 10.3109/10428199309149129

[22] Armstrong JK, Hempel G, Koling S, , Antibody against poly(ethylene glycol) adversely affects PEG-asparaginase therapy in acute lymphoblastic leukemia patients, Cancer 110 (1) (2007) 103–111. 17516438 10.1002/cncr.22739

[23] Garay RP, El-Gewely R, Armstrong JK, Garratty G, Richette P, Antibodies against polyethylene glycol in healthy subjects and in patients treated with PEG-conjugated agents, Expet Opin. Drug Deliv. 9 (11) (2012) 1319–1323. 10.1517/17425247.2012.720969 22931049

[24] Tong WH, Pieters R, Kaspers GJ, , A prospective study on drug monitoring of PEGasparaginase and Erwinia asparaginase and asparaginase antibodies in pediatric acute lymphoblastic leukemia, Blood 123 (13) (2014) 2026–2033. 24449211 10.1182/blood-2013-10-534347 PMC3968389

[25] Panosyan EH, Seibel NL, Martin-Aragon S, , Asparaginase antibody and asparaginase activity in children with higher-risk acute lymphoblastic leukemia: children’s Cancer Group Study CCG-1961, J. Pediatr. Hematol. Oncol. 26 (4) (2004) 217–226. 15087948 10.1097/00043426-200404000-00002

[26] Burke MJ, How to manage asparaginase hypersensitivity in acute lymphoblastic leukemia, Future Oncol. 10 (16) (2014) 2615–2627. 24983955 10.2217/fon.14.138

[27] van der Sluis IM, Vrooman LM, Pieters R, , Consensus expert recommendations for identification and management of asparaginase hypersensitivity and silent inactivation, Haematologica 101 (3) (2016) 279–285. 26928249 10.3324/haematol.2015.137380 PMC4815719

[28] Mondelaers V, Ferster A, Uyttebroeck A, , Prospective, real-time monitoring of pegylated Escherichia coli and Erwinia asparaginase therapy in childhood acute lymphoblastic leukaemia and non-Hodgkin lymphoma in Belgium, Br. J. Haematol. 190 (1) (2020) 105–114. 32057100 10.1111/bjh.16495

[29] Kodera Y, Sekine T, Yasukohchi T, , Stabilization of L-asparaginase modified with comb-shaped poly(ethylene glycol) derivatives, in vivo and in vitro, Bioconjugate Chem. 5 (4) (1994) 283–286. 10.1021/bc00028a001 7948093

[30] Wang B, Cao Y, Chi S, Lou D, A PEGylation technology of L-asparaginase with monomethoxy polyethylene glycol-propionaldehyde, Z. Naturforsch., C: J. Biosci. 67 (5–6) (2012) 312–318. 22888537 10.1515/znc-2012-5-611

[31] Van Trimpont M, Schalk AM, De Visser Y, , In vivo stabilization of a less toxic asparaginase variant leads to a durable anti-tumor response in acute leukemia, Haematologica 108 (2) (2022) 409–419. 10.3324/haematol.2022.281390 PMC9890011 35979719

[32] Schmidt MP, Ivanov AV, Coriu D, Miron IC, L-asparaginase toxicity in the treatment of children and adolescents with acute lymphoblastic leukemia, J. Clin. Med. 10 (19) (2021). 10.3390/jcm10194419 PMC8509606 34640436

[33] Schmiegelow K, Rank CU, Stock W, Dworkin E, van der Sluis I, SOHO state of the art updates and next questions: management of asparaginase toxicity in adolescents and young adults with acute lymphoblastic leukemia, Clin. Lymphoma, Myeloma & Leukemia 21 (11) (2021) 725–733. 10.1016/j.clml.2021.07.009 34511319

[34] Noguchi K, Ikawa Y, Takenaka M, , L-asparaginase as an efficient salvage therapy for refractory acute myeloid leukemia with chromosome 7 abnormalities: a case series, Int. J. Hematol. 118 (3) (2023) 406–410. 37022561 10.1007/s12185-023-03591-1

[35] Emadi A, Kapadia B, Bollino D, , Venetoclax and pegcrisantaspase for complex karyotype acute myeloid leukemia, Leukemia 35 (7) (2021) 1907–1924. 33199836 10.1038/s41375-020-01080-6 PMC10976320

[36] Bertuccio SN, Serravalle S, Astolfi A, , Identification of a cytogenetic and molecular subgroup of acute myeloid leukemias showing sensitivity to L-Asparaginase, Oncotarget 8 (66) (2017) 109915–109923. 29299118 10.18632/oncotarget.18565 PMC5746353

[37] Lorenzi PL, Weinstein JN, Asparagine synthetase: a new potential biomarker in ovarian cancer, Drug News Perspect. 22 (1) (2009) 61–64. 19209300 10.1358/dnp.2009.22.1.1303820 PMC4096155

[38] Yu M, Henning R, Walker A, , L-asparaginase inhibits invasive and angiogenic activity and induces autophagy in ovarian cancer, J. Cell Mol. Med. 16 (10) (2012) 2369–2378. 22333033 10.1111/j.1582-4934.2012.01547.x PMC3416969

[39] Hays JL, Kim G, Walker A, , A phase II clinical trial of polyethylene glycol-conjugated L-asparaginase in patients with advanced ovarian cancer: early closure for safety, Mol. Clin. Oncol. 1 (3) (2013) 565–569. 24649212 10.3892/mco.2013.99 PMC3916154

[40] Blachier J, Cleret A, Guerin N, , L-asparaginase anti-tumor activity in pancreatic cancer is dependent on its glutaminase activity and resistance is mediated by glutamine synthetase, Exp. Cell Res. 426 (2) (2023) 113568. 36967104 10.1016/j.yexcr.2023.113568

[41] Dufour E, Gay F, Aguera K, , Pancreatic tumor sensitivity to plasma L-asparagine starvation, Pancreas 41 (6) (2012) 940–948. 22513289 10.1097/MPA.0b013e318247d903

[42] Bachet JB, Gay F, Marechal R, , Asparagine synthetase expression and phasé I study with L-asparaginase encapsulated in red blood cells in patients with pancreatic adenocarcinoma, Pancreas 44 (7) (2015) 1141–1147. 26355551 10.1097/MPA.0000000000000394

[43] Zhang B, Dong LW, Tan YX, , Asparagine synthetase is an independent predictor of surgical survival and a potential therapeutic target in hepatocellular carcinoma, Br. J. Cancer 109 (1) (2013) 14–23. 23764751 10.1038/bjc.2013.293 PMC3708586

[44] Bai J, Tang R, Zhou K, , An asparagine metabolism-based classification reveals the metabolic and immune heterogeneity of hepatocellular carcinoma, BMC Med. Genom. 15 (1) (2022) 222. 10.1186/s12920-022-01380-z PMC9594908 36284275

[45] Anaya JM, Shoenfeld Y, Rojas-Villarraga A, Levy RA, Cervera R (Eds.), Autoimmunity: from Bench to Bedside, Bogota (Colombia): El Rosario University Press © 2013 Universidad del Rosario, 2013. 29087650

[46] Hinze L, Pfirrmann M, Karim S, , Synthetic lethality of wnt pathway activation and asparaginase in drug-resistant acute leukemias, Cancer Cell 35 (4) (2019) 664–676.e667. 30991026 10.1016/j.ccell.2019.03.004 PMC6541931

[47] Hinze L, Labrosse R, Degar J, , Exploiting the therapeutic interaction of WNT pathway activation and asparaginase for colorectal cancer therapy, Cancer Discov. 10 (11) (2020) 1690–1705. 32703769 10.1158/2159-8290.CD-19-1472 PMC7642035

[48] Du F, Chen J, Liu H, , SOX12 promotes colorectal cancer cell proliferation and metastasis by regulating asparagine synthesis, Cell Death Dis. 10 (3) (2019) 239. 30858360 10.1038/s41419-019-1481-9 PMC6412063

[49] Knott SRV, Wagenblast E, Khan S, , Asparagine bioavailability governs metastasis in a model of breast cancer, Nature 554 (7692) (2018) 378–381. 29414946 10.1038/nature25465 PMC5898613

[50] Nguyen HA, Su Y, Lavie A, Design and characterization of Erwinia chrysanthemi l-asparaginase variants with diminished l-glutaminase activity, J. Biol. Chem. 291 (34) (2016) 17664–17676. 27354283 10.1074/jbc.M116.728485 PMC5016162

[51] Nguyen HA, Su Y, Zhang JY, , A novel l-asparaginase with low l-glutaminase coactivity is highly efficacious against both T- and B-cell acute lymphoblastic leukemias in vivo, Cancer Res. 78 (6) (2018) 1549–1560. 29343523 10.1158/0008-5472.CAN-17-2106 PMC5856643

[52] Schalk AM, Nguyen HA, Rigouin C, Lavie A, Identification and structural analysis of an L-asparaginase enzyme from Guinea pig with putative tumor cell killing properties, J. Biol. Chem. 289 (48) (2014) 33175–33186. 25320094 10.1074/jbc.M114.609552 PMC4246078

[53] Fernandez CA, Cai X, Elozory A, , High-throughput asparaginase activity assay in serum of children with leukemia, Int. J. Clin. Exp. Med. 6 (7) (2013) 478–487. 23936585 PMC3731178

[54] Ollenschläger G, Roth E, Linkesch W, Jansen S, Simmel A, Mödder B, Asparaginase-induced derangements of glutamine metabolism: the pathogenetic basis for some drug-related side-effects, Eur. J. Clin. Invest. 18 (5) (1988) 512–516. 3147904 10.1111/j.1365-2362.1988.tb01049.x

[55] Reinert RB, Oberle LM, Wek SA, , Role of glutamine depletion in directing tissue-specific nutrient stress responses to L-asparaginase, J. Biol. Chem. 281 (42) (2006) 31222–31233. 16931516 10.1074/jbc.M604511200

[56] Grima-Reyes M, Vandenberghe A, Nemazanyy I, , Tumoral microenvironment prevents de novo asparagine biosynthesis in B cell lymphoma, regardless of ASNS expression, Sci. Adv. 8 (27) (2022) eabn6491. 35857457 10.1126/sciadv.abn6491 PMC9258813

[57] Phillipson-Weiner L, Mirek ET, Wang Y, McAuliffe WG, Wek RC, Anthony TG, General control nonderepressible 2 deletion predisposes to asparaginase-associated pancreatitis in mice, Am. J. Physiol. Gastrointest. Liver Physiol. 310 (11) (2016) G1061–G1070. 26968207 10.1152/ajpgi.00052.2016 PMC4935488

[58] Warrell RP, Arlin ZA, Gee TS, Chou TC, Roberts J, Young CW, Clinical evaluation of succinylated Acinetobacter glutaminase-asparaginase in adult leukemia, Cancer Treat Rep. 66 (7) (1982) 1479–1485. 7046929

[59] Holcenberg JS, Borella LD, Camitta BM, Ring BJ, Human pharmacology and toxicology of succinylated Acinetobacter glutaminase-asparaginase, Cancer Res. 39 (8) (1979) 3145–3151. 455299

[60] Warrell RP, Chou TC, Gordon C, , Phase I evaluation of succinylated Acinetobacter glutaminase-asparaginase in adults, Cancer Res. 40 (12) (1980) 4546–4551. 7438089

[61] Durden DL, Salazar AM, Distasio JA, Kinetic analysis of hepatotoxicity associated with antineoplastic asparaginases, Cancer Res. 43 (4) (1983) 1602–1605. 6339039

[62] Durden DL, Distasio JA, Characterization of the effects of asparaginase from Escherichia coli and a glutaminase-free asparaginase from Vibrio succinogenes on specific ell-mediated cytotoxicity, Int. J. Cancer 27 (1) (1981) 59–65. 7019106 10.1002/ijc.2910270110

[63] Distasio JA, Salazar AM, Nadji M, Durden DL, Glutaminase-free asparaginase from vibrio succinogenes: an antilymphoma enzyme lacking hepatotoxicity, Int. J. Cancer 30 (3) (1982) 343–347. 6752048 10.1002/ijc.2910300314

[64] Villa P, Corada M, Bartosek I, L-asparaginase effects on inhibition of protein synthesis and lowering of the glutamine content in cultured rat hepatocytes, Toxicol. Lett. 32 (3) (1986) 235–241. 3535170 10.1016/0378-4274(86)90113-x

[65] Chiu M, Tardito S, Pillozzi S, , Glutamine depletion by crisantaspase hinders the growth of human hepatocellular carcinoma xenografts, Br. J. Cancer 111 (6) (2014) 1159–1167. 25072259 10.1038/bjc.2014.425 PMC4453854

[66] Long S, Zhang X, Rao Z, , Amino acid residues adjacent to the catalytic cavity of tetramer l-asparaginase II contribute significantly to its catalytic efficiency and thermostability, Enzym. Microb. Technol. 82 (2016) 15–22. 10.1016/j.enzmictec.2015.08.009 26672444

[67] Offman MN, Krol M, Patel N, , Rational engineering of L-asparaginase reveals importance of dual activity for cancer cell toxicity, Blood 117 (5) (2011) 1614–1621. 21106986 10.1182/blood-2010-07-298422

[68] Parmentier JH, Maggi M, Tarasco E, Scotti C, Avramis VI, Mittelman SD, Glutaminase activity determines cytotoxicity of L-asparaginases on most leukemia cell lines, Leuk. Res. 39 (7) (2015) 757–762. 25941002 10.1016/j.leukres.2015.04.008 PMC4458142

[69] Maggi M, Chiarelli LR, Valentini G, Scotti C, Engineering of Helicobacter pylori L-asparaginase: characterization of two functionally distinct groups of mutants, PLoS One 10 (2) (2015) e0117025. 25664771 10.1371/journal.pone.0117025 PMC4321988

[70] Chan WK, Horvath TD, Tan L, , Glutaminase activity of L-asparaginase contributes to durable preclinical activity against acute lymphoblastic leukemia, Mol. Cancer Therapeut. 18 (9) (2019) 1587–1592. 10.1158/1535-7163.MCT-18-1329 PMC6726508 31209181

[71] Chan WK, Lorenzi PL, Anishkin A, , The glutaminase activity of L-asparaginase is not required for anticancer activity against ASNS-negative cells, Blood 123 (23) (2014) 3596–3606. 24659632 10.1182/blood-2013-10-535112 PMC4047499

[72] Apfel V, Begue D, Cordo V, , Therapeutic assessment of targeting ASNS combined with l-asparaginase treatment in solid tumors and investigation of resistance mechanisms, ACS Pharmacol. Transl. Sci. 4 (1) (2021) 327–337. 33615182 10.1021/acsptsci.0c00196 PMC7887857

[73] Li H, Ning S, Ghandi M, , The landscape of cancer cell line metabolism, Nat. Med. 25 (5) (2019) 850–860. 31068703 10.1038/s41591-019-0404-8 PMC6629041

[74] Nakamura A, Nambu T, Ebara S, , Inhibition of GCN2 sensitizes ASNS-low cancer cells to asparaginase by disrupting the amino acid response, Proc. Natl. Acad. Sci. U.S.A. 115 (33) (2018) E7776–e7785. 30061420 10.1073/pnas.1805523115 PMC6099884

[75] Faschinger AM, Sessler N, Development of a lyophilized formulation of pegaspargase and comparability versus liquid pegaspargase, Adv. Ther. 36 (8) (2019) 2106–2121. 31140125 10.1007/s12325-019-00988-5 PMC6822849

[76] Narta UK, Kanwar SS, Azmi W, Pharmacological and clinical evaluation of L-asparaginase in the treatment of leukemia, Crit. Rev. Oncol. Hematol. 61 (3) (2007) 208–221. 17011787 10.1016/j.critrevonc.2006.07.009

