# Supplementary material for: A human-like glutaminase-free asparaginase is highly efficacious in ASNSlow leukemia and solid cancer mouse xenograft models
Source: Cancer Lett. Author manuscript; Available in PMC 2026 Jun 19. (PMC12177098; doi:10.1016/j.canlet.2024.217404)
Supplement: Supplemental [file NIHMS2083833-supplement-Supplemental.pdf]

<u>Supplemental Data</u>

**Table S1. Amino acid identity to the human asparaginase homolog (*h*A)**	
**Full length *Gp*A WT**	69.8 %
***Gp*A369**	72.2 %
**EBD-200**	83.0 %

Fig. S1

A

Protein sample	Source	Km (mM)	kcat (sec^{-1})
E. coli L-ASNase WT	Lorenzi lab	0.012	32
E. coli L-ASNase Q59L	Lorenzi lab	0.32	15.6
E. coli L-ASNase WT	Lavie lab	0.015	44
E. coli L-ASNase Q59L	Lavie lab	1.4 – 1.7	1.1 – 1.3

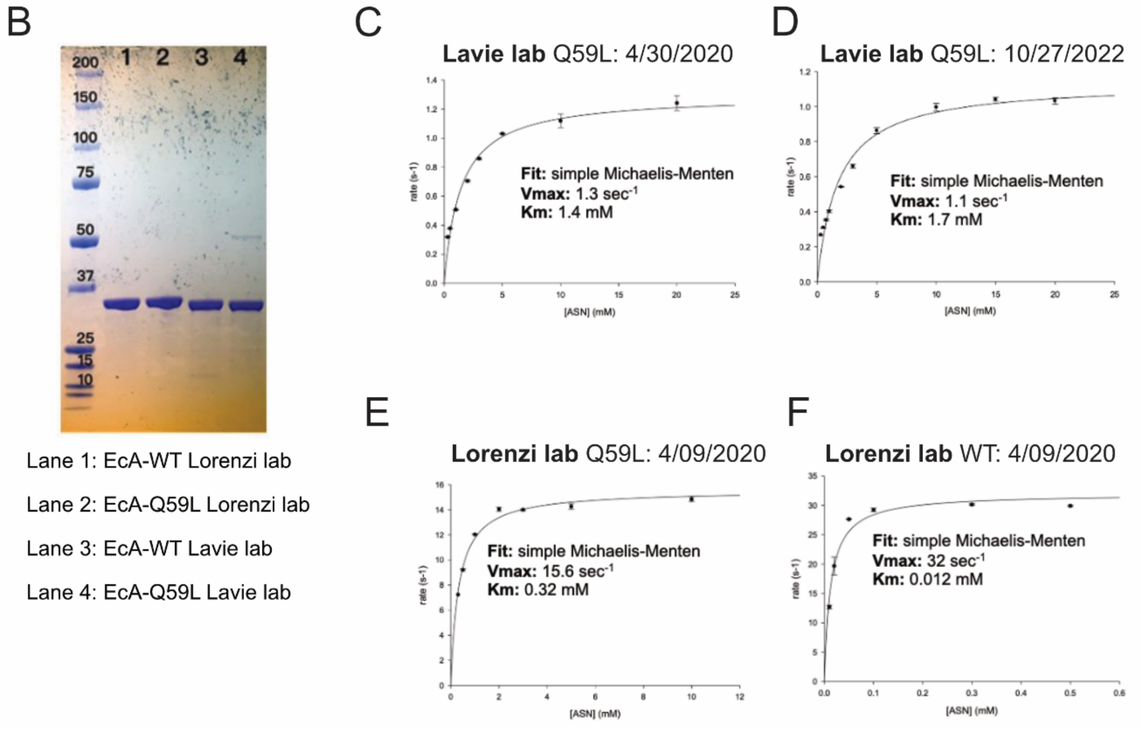

Figure S1: Kinetic data and SDS-PAGE for E. coli WT and Q59L, made by us and gifted. (A) *Summary of kinetic parameters for Lavie and Lorenzi Labs E. coli L-ASNase* ***(B)*** *SDS-PAGE of Lavie and Lorenzi Labs E. coli L-ASNase. The slightly higher apparent molecular weight of the Lorenzi Lab enzymes is due to the presences of a C-terminal his-tag. The N-terminal periplasmic localization sequence present in the clone is cleaved during the transport to the periplasm, so is not in the final protein. The endogenous E. coli L-ASNase is also processed in this way. In contrast, the Lavie enzymes were produced as His-SUMO fusion proteins that remained in the cytoplasm. The His-SUMO tag was cleaved and the proteins further purified by gel filtration.* ***(C-F)*** *Fit of steady state kinetic data to the Michaelis-Menten equation. The large discrepancy between our (Lavie Lab) kinetic parameters and the gifted Q59L (Lorenzi Lab) prompted us to completely redo the expression, purification, and kinetic analysis. As a result, shown are the data from the Q59L study done on 04/30/2020 and the confirmatory study done on 10/27/2022.*

Fig. S2

A

Protein sample	Source	Ratio	WT:Q59L
E. coli L-ASNase WT	Lavie lab	1	0
E. coli L-ASNase WT	Lorenzi lab	1	0
E. coli L-ASNase Q59L	Lavie lab	1	160
E. coli L-ASNase Q59L	Lorenzi lab	3	1

B

C

D

E

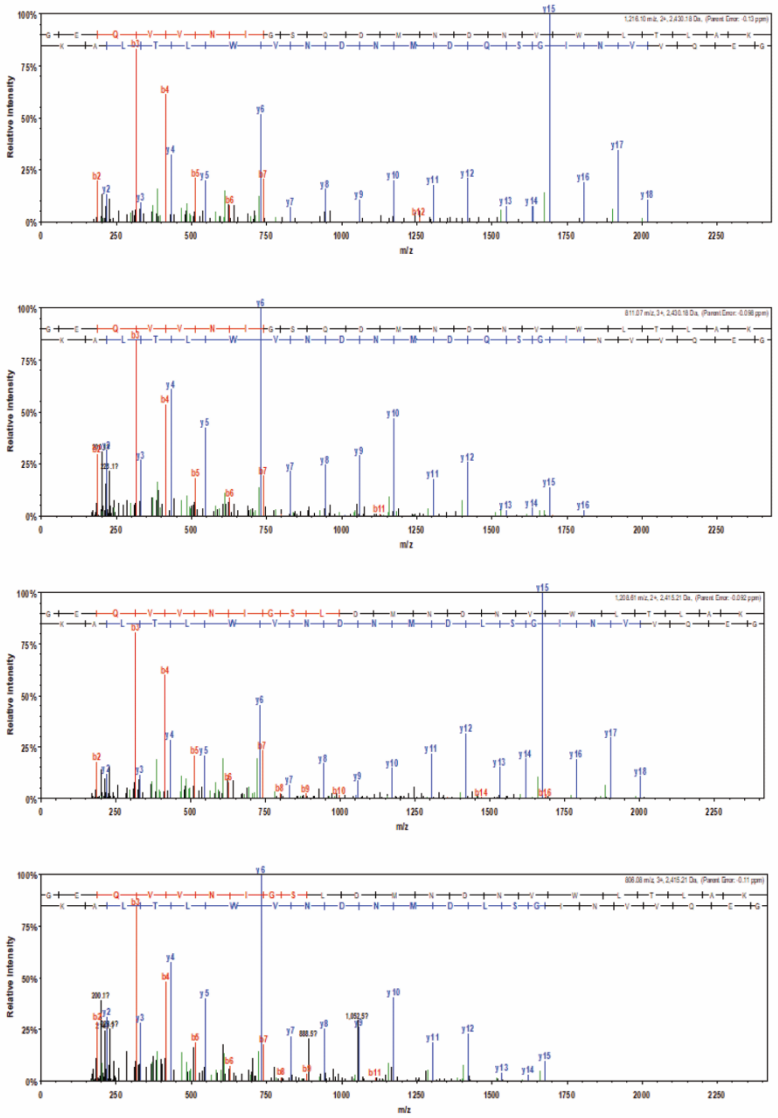

***Figure S2. Mass Spectrometry analysis of WT and Q59L E. coli variants produced by the Lavie and Lorenzi Labs. (A)** Summary of WT:Q59L protein ratio in the samples. **(B)** MS/MS of P1 in charge state 2+. **(C)** MS/MS of P1 in charge state of 3+. **(D)** MS/MS of P2 in charge state of 2+. **(E)** MS/MS of P2 in charge state of 3+.*

Fig. S3

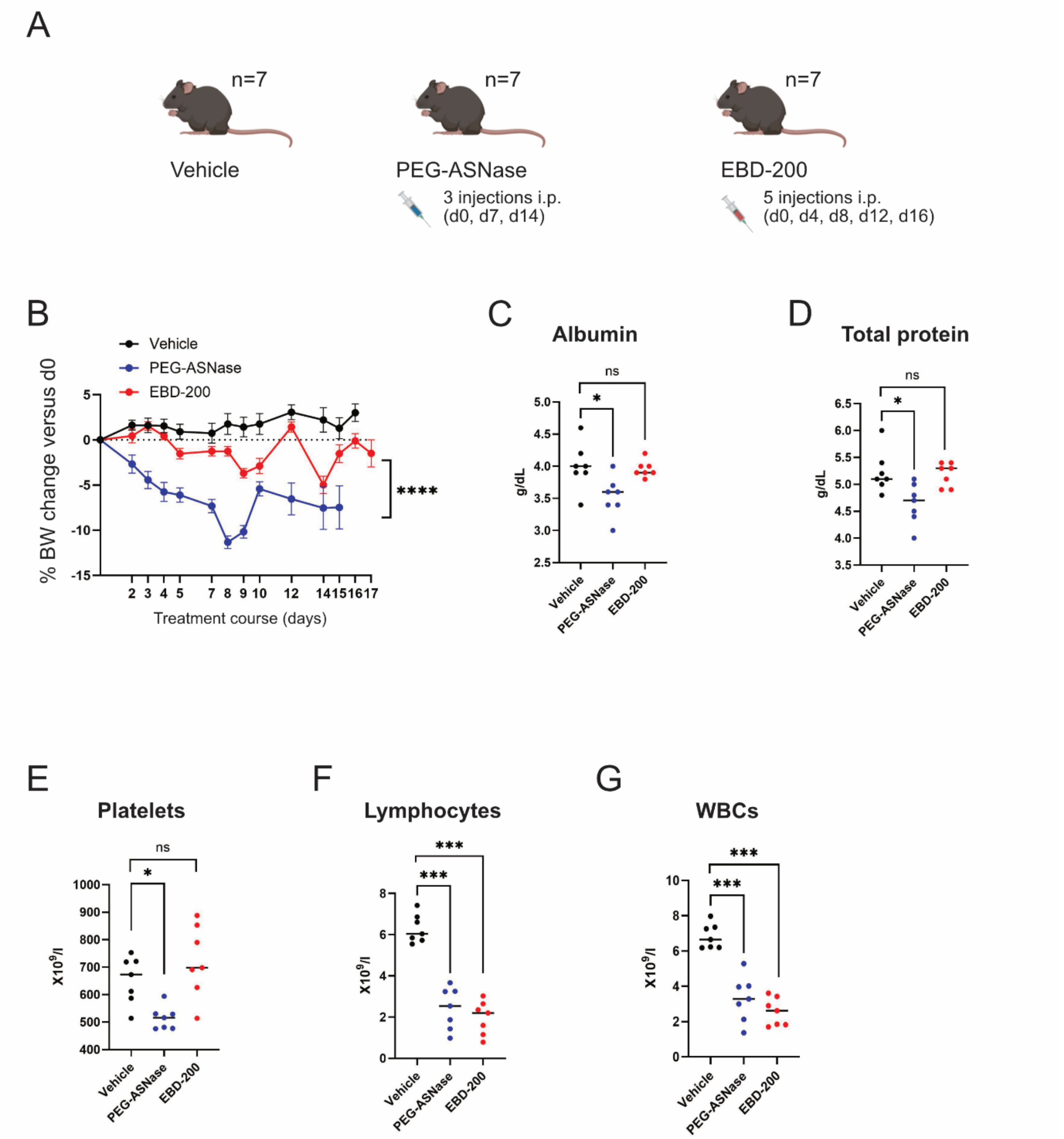

Figure S3. Toxicity profiling of EBD-200. (A) *Experimental set-up of the repeated dose toxicity study. PEG-ASNase was administered every 7 days via intraperitoneal injection (3000 IU/kg; total of 3 shots), while EBD-200 was administered every 4 days (3000 IU/kg; total of 5 shots).* ***(B)*** *Mice treated with PEG-ASNase loose significantly more weight during treatment compared to EBD-200 treated mice (p <0.0001; Mixed Effects Model). Mean with SEM is plotted.* ***(C-G)*** *Repeated dosing of PEG-ASNase has a significant effect on albumin (p = 0.0006; Mann-Whitney test), total protein (p = 0.0128; Mann-Whitney test) and platelets (p = 0.0111; Mann-Whitney test). However, no significant changes in blood platelets, albumin or total protein were observed for EBD-200. Remarkably, there is a significant decrease in white blood cells and lymphocytes (p = 0.0006; Mann-Whitney test), which would suggest that this observed toxicity is not related to the co-glutaminase activity.*

Fig. S4

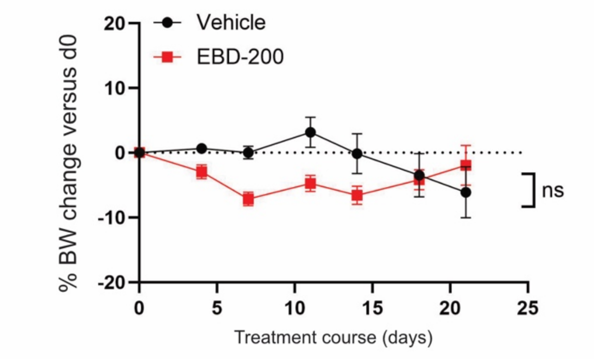

Figure S4. Body weight change of EBD-200 treated mice in the melanoma ASNS-KO model. *Body weights of the mice were carefully monitored during treatment and shows that there is no significant difference in body weights when comparing vehicle and EBD-200 treated mice was observed (p >0.05; Mixed Effects Model). Mean with SEM is plotted.*

Fig. S5

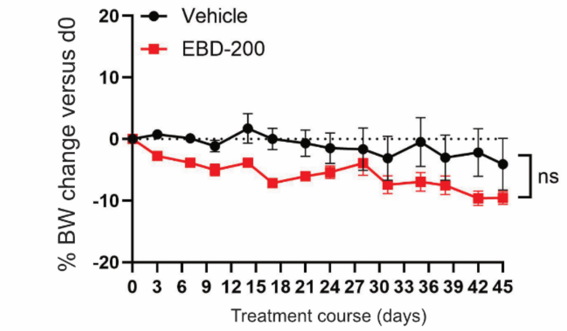

Figure S5. Body weight change of EBD-200 treated mice in the JHH5-luc liver cancer model. *Body weights of the mice were carefully monitored during treatment and shows that there is no significant difference in body weights when comparing vehicle and EBD-200 treated mice was observed (p >0.05; Mixed Effects Model). Mean with SEM is plotted.*
